# Current Use, Monitoring and Reversal of Neuromuscular Block in Pediatric Anesthesia and Intensive Care: A Web‐Based Cross‐Sectional ESAIC Survey

**DOI:** 10.1002/pan.70285

**Published:** 2026-08-15

**Authors:** Giulia Bonatti, Mirjana Gander, Arash Afshari, Francis Veyckemans, Thomas Fuchs‐Buder, Thomas Riva, Markus Huber, Nicola Disma, Alexander Fuchs

**Affiliations:** ^1^ Unit for Research in Anaesthesia IRCCS Istituto Giannina Gaslini Genoa Italy; ^2^ Department of Anaesthesiology and Pain Medicine, Inselspital, Bern University Hospital University of Bern Bern Switzerland; ^3^ Department of Paediatric and Obstetric Anaesthesia, Juliane Marie Centre, Rigshospitalet & Institute of Clinical Medicine University of Copenhagen Copenhagen Denmark; ^4^ UCLouvain Medical School Brussels Belgium; ^5^ Université de Lorraine CHRU Nancy, Hôpital de Brabois Nancy France

**Keywords:** neuromuscular block, neuromuscular monitoring, pediatric anesthesia, residual neuromuscular block, rocuronium, RSI, sugammadex, survey study, TOF, train‐of‐four monitoring

## Abstract

**Background:**

Neuromuscular blocking agents are recommended by the European Society of Anaesthesiology and Intensive Care (ESAIC) and European Society of Paediatric Anaesthesia (ESPA) for facilitating tracheal intubation in children. Despite these recommendations, real‐world data on how neuromuscular blocking agents are used, monitored, and reversed remain scarce.

**Aims:**

To identify and characterize self‐reported neuromuscular blocking agent practice patterns across European and non‐European countries, in the period preceding publication of the joint ESAIC and ESPA guidelines.

**Methods:**

We conducted an anonymous, cross‐sectional survey between 12 November and 12 December 2025, targeting members of the ESAIC, ESPA, and the French and Spanish national pediatric anesthesiology societies involved in pediatric airway management across 43 countries.

**Results:**

The survey was accessed by 904 persons, and 874 responses were included in the analysis. Most participants were from Europe (83.5%) and worked in mixed pediatric anesthesia and intensive care settings (71.2%). Rocuronium was the most frequently used neuromuscular blocking agent for both routine tracheal intubation (68%; 95% CI 65%–71%) and rapid sequence intubation (67%; 95% CI 64%–70%). Neuromuscular blocking agents were used “always” or “mostly” before routine intubation in 47% of neonates (≤ 30 days) and 50% of infants (< 1 year), whereas “rarely” or “never” use was reported in 30% and 24%, respectively. Most respondents (56%) assessed adequacy of neuromuscular block using a fixed time interval after neuromuscular blocking agent administration. Reversal was administered “always” before extubation by 27% of respondents, while 9.3% reported “rarely” and 2.9% “never.” Only half of participants used quantitative neuromuscular monitoring to guide reversal. Sugammadex was used by 47%.

**Conclusions:**

Self‐reported clinical practice regarding neuromuscular blocking agent use in pediatric patients remains heterogeneous. Children are frequently intubated without neuromuscular blocking agents and extubated without consistent reversal. Quantitative neuromuscular monitoring remains uncommon. These practice patterns raise concerns for patient safety.

## Clinical Implications

**What is already known about the topic?:**

○ Neuromuscular blocking agents facilitate tracheal intubation in children and are recommended by the guideline.
○ Real‐world practice among European pediatric anesthetists and intensivists prior to guideline publication had not previously been described.

**What new information this study adds?:**

○ This large multinational survey shows that pre‐guideline clinical practice was highly heterogeneous.
○ Children were frequently intubated without neuromuscular blocking agents, quantitative monitoring was used by only half of respondents, and consistent reversal before extubation was reported by fewer than one in three clinicians.
○ These findings identify critical education and training gaps that must be addressed to support safe and effective implementation of the new guidelines.

## 1 Introduction

Neuromuscular blocking agents (NMBA) are widely used in anesthetic practice to induce neuromuscular block (NMB) and facilitate tracheal intubation and optimize surgical conditions [1, 2]. The European Society of Anaesthesiology and Intensive Care (ESAIC) and British Journal of Anaesthesia (BJA) airway management guidelines for infants and neonates as well as the most recent ESAIC and European Society of Paediatric Anaesthesia (ESPA) guidelines on neuromuscular block in anesthetized children recommend to achieve NMB before tracheal intubation [3, 4, 5]. Pediatric anesthesia encompasses a broad age spectrum, from neonates to adolescents, with substantial physiological and pharmacological heterogeneity influencing NMBA pharmacokinetics/pharmacodynamics and NMB recovery profiles [6]. Short procedure durations or redosing requirements, and children's anatomical vulnerabilities increase the risk of residual NMB and associated perioperative respiratory complications [7, 8]. Despite technical advances in quantitative NMB monitoring and reversal strategies (e.g., the availability of sugammadex to reverse steroid NMBA), residual NMB remains clinically relevant in children [8], and current practice patterns for NMBA choice, NMB monitoring, and reversal remain poorly characterized. The 2025 Guidelines on pediatric neuromuscular block provide clear recommendations on how to improve clinical practice [9].

The primary aim of this study was to describe current clinical practices regarding NMBA selection, NMB monitoring strategies, and reversal approaches in pediatric anesthesia and intensive care prior to publication of the 2025 Guidelines on pediatric neuromuscular block [5]. Secondary aims were to assess how these practice patterns vary with patient age, anesthetist's experience, hospital setting, and geographical region.

This baseline survey provides essential data to identify targets for implementation and enable future assessments of guideline impact.

## 2 Materials and Methods

### 2.1 Ethics Approval

The survey protocol was submitted to the Cantonal Ethics Committee of Bern, which waived the requirement for formal ethical approval in accordance with Swiss human research legislation (Req‐2025‐01277, 22 September 2025, Chairperson: Prof. em. Dr. med. Martin Fey). Participation was entirely voluntary and uncompensated. The survey was fully anonymous, with no collection of personal identifiers. Respondents provided implied consent by accessing, completing, and submitting the questionnaire.

### 2.2 Study Design, Setting, and Participants

We conducted an international, anonymous, cross‐sectional, web‐based survey to capture current clinical practice patterns regarding NMB in pediatric anesthesia. The survey was officially endorsed and launched on 12th November 2025 by the ESAIC to all its members. It was subsequently re‐launched by three large pediatric anesthesia societies: the ESPA, the French “Association des Anesthésistes‐Réanimateurs Pédiatriques d'Expression Française (ADARPEF),” and the Spanish “Sociedad Española de Anestesiología, Reanimación y Terapéutica del Dolor (SEDAR).”

Dissemination occurred through society mailing lists, institutional networks, and social media channels to maximize reach among practicing anesthesiologists. Eligible participants were clinicians with full or mixed pediatric anesthesia practice working in academic, district or private hospitals across European and non‐European countries. The sole inclusion criterion was current involvement in pediatric anesthesia; no further restrictions were applied regarding seniority, subspecialty, or geography. The survey remained open until 12th December 2025.

### 2.3 Questionnaire Development and Data Collection

All data were self‐reported via a structured online questionnaire hosted on SurveyMonkey (San Mateo, CA, USA). The questionnaire encompassed key domains relevant to NMB practice: respondent demographics and practice setting; preferred NMBAs for intubation; availability and frequency use of neuromuscular monitoring; reversal strategies; and awareness of current regulatory restrictions on pediatric sugammadex use.

Prior to launch, the questionnaire was piloted by 14 physicians from Denmark, France, Germany, Italy and Switzerland who had no role in its development. This pilot phase evaluated clarity, relevance, logical flow, and completion time (typically 8–12 min), resulting in minor wording refinements for precision and respondent‐friendliness. Platform‐standard measures ensured anonymity, including IP‐based duplicate prevention and no storage of identifiable data.

### 2.4 Outcomes and Variables

Primary outcomes focused on NMBA selection and use patterns. These included the most reported NMBA for tracheal intubation in distinct clinical scenarios: routine intubation, rapid sequence induction and intubation (RSII) with predicted normal airway and risk of pulmonary aspiration. We also assessed self‐reported frequency of NMBA administration prior to routine intubation, stratified by pediatric age group (neonates, infants, and children ≥ 1 year).

Secondary outcomes addressed neuromuscular block monitoring (availability, frequency of use overall and by patient age/anatomical site) and reversal practices (presence of local guidance, choice of agent, administration frequency, typical sugammadex doses for deep versus moderate block). Respondents also reported any restrictions on pediatric sugammadex use, categorized as institutional policies, age‐based limitations, cost or efficacy concerns, regulatory labelling, or other free‐text reasons. Covariates included respondent experience (years of independent practice), hospital type, case volume, and country or region.

For questions on age‐related neuromuscular monitoring practices, respondents were asked about the age categories of > 1 month, > 1 year, and > 3 years. These categories were selected pragmatically during questionnaire development because they align with commonly recognized developmental stages and are frequently used in pediatric anesthesia practice.

### 2.5 Bias and Sample Size

We acknowledge potential sources of bias inherent in survey research, including self‐selection bias (where respondents may be more motivated due to personal interest in the topic) and inaccuracies in self‐reported practice patterns, which may not fully reflect actual clinical behaviors. No adjustments for non‐response bias (e.g., weighting or imputation) were performed because denominator data for the total number of invitees were unavailable due to the open dissemination strategy.

No formal sample size calculation was performed for this explorative survey. Instead, all eligible respondents who accessed the survey, completed key items, and submitted their responses during the study period were included, yielding a pragmatic sample reflective of engaged pediatric anesthesia and intensive care clinicians from European and non‐European regions.

### 2.6 Statistical Analysis

Ordinal responses (e.g., frequency scales from “never” to “always”) were numerically coded from 0 to 5 for analysis. Years of independent practice were categorized as follows: in training, 0–5 years, 6–10 years, 11–25 years, or > 25 years. Results are presented as frequencies, percentages, and 95% confidence intervals (CI).

Between‐group differences for ordinal frequency scales (e.g., across age groups) were evaluated using the Friedman non‐parametric test, with a two‐sided *p* < 0.05 denoting statistical significance. All analyses adopted a complete‐case approach per outcome, with denominators explicitly reported to account for partial non‐response. Reporting adheres to STROBE (Strengthening the Reporting of Observational Studies in Epidemiology) guidelines for cross‐sectional studies [10].

## 3 Results

A total of 904 clinicians accessed the survey, and 874 respondents completed at least the demographic section and were included in the analysis. The majority of survey participants were geographically located in Europe (83.5%, *n* = 730), with France (12.5%, *n* = 109), the United Kingdom (7.9%, *n* = 69), Germany (7.6%, *n* = 66), and Spain (6.3%, *n* = 55) representing the largest contribution (Figure 1). Participants represented a wide range of professional experience: 19.5% had 0–5 years of experience, 20.6% had 6–10 years, 38.4% had 11–25 years, and 16.0% had more than 25 years (Table 1). Most survey participants reported working in mixed pediatric anesthesia and intensive care settings (71.2%), while 19.6% worked exclusively in pediatric anesthesia, and 6.2% exclusively in pediatric intensive care. Accordingly, the respondents reported performing airway management most frequently in the operating room (97%), but a substantial proportion of respondents also reported performing airway management in non‐operating room locations (41%), emergency departments (33%), intensive care units (28%), and pre‐hospital settings (10%).

**FIGURE 1:**
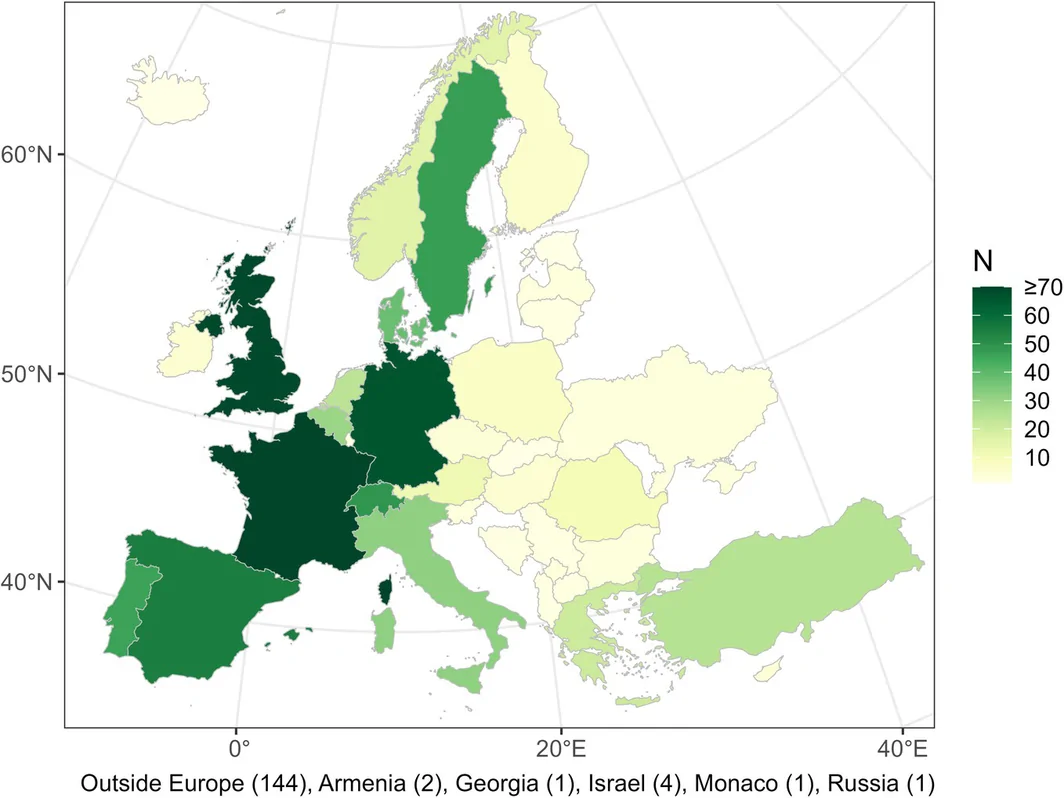
Geographical distribution of European survey participants.

**TABLE 1:** Survey respondents' baseline characteristics.

All	*N* = 874
Please indicate your main area of practice	
Exclusively pediatric anesthesia	171 (19.6%)
Mixed adult and pediatric anesthesia	7 (0.8%)
Mixed pediatric intensive care unit and pediatric anesthesia	622 (71.2%)
Exclusively pediatric intensive care unit	54 (6.2%)
Other (please specify)	20 (2.3%)
Your age group	
20–30	38 (4.3%)
31–40	274 (31.4%)
41–50	305 (34.9%)
51–60	156 (17.8%)
> 60	90 (10.3%)
Prefer not to answer	11 (1.3%)
Your gender identity	
Female	428 (49.0%)
Male	430 (49.2%)
Non‐binary	2 (0.2%)
Other/prefer to self‐describe	2 (0.2%)
Prefer not to answer	12 (1.4%)
Number of years of independent anesthetic practice	
Currently in training	48 (5.5%)
0–5	170 (19.5%)
6–10	180 (20.6%)
11–25	336 (38.4%)
> 25	140 (16.0%)
Country of primary practice	
Europe	730 (83.5%)
Outside of Europe	144 (16.5%)
Main primary practice setting	
University/teaching hospital	598 (68.4%)
Non‐academic hospital/clinic	276 (31.6%)

*Note:* Data are presented as number (percentage).

### 3.1 Neuromuscular Blocking Agents for Routine Pediatric Tracheal Intubation

Among 874 survey participants, rocuronium was the most used NMBA for routine pediatric tracheal intubation (68%; 95% CI 65–71), followed by atracurium (13%; 95% CI 11–15). Succinylcholine was rarely selected as NMBA for routine intubation (4.9%; 95% CI 3.6–6.6) (Figure 2). The reported frequency of NMBA use before routine intubation varied across age groups. NMBA were used “always” or “mostly” in 47% of neonates (≤ 30 days), 50% of infants (< 1 year), and 52% of children (≥ 1 year). Conversely, “rarely” or “never” use was reported in 30% of neonates, 24% of infants, and 15% of children ≥ 1 year (Table 2).

**FIGURE 2:**
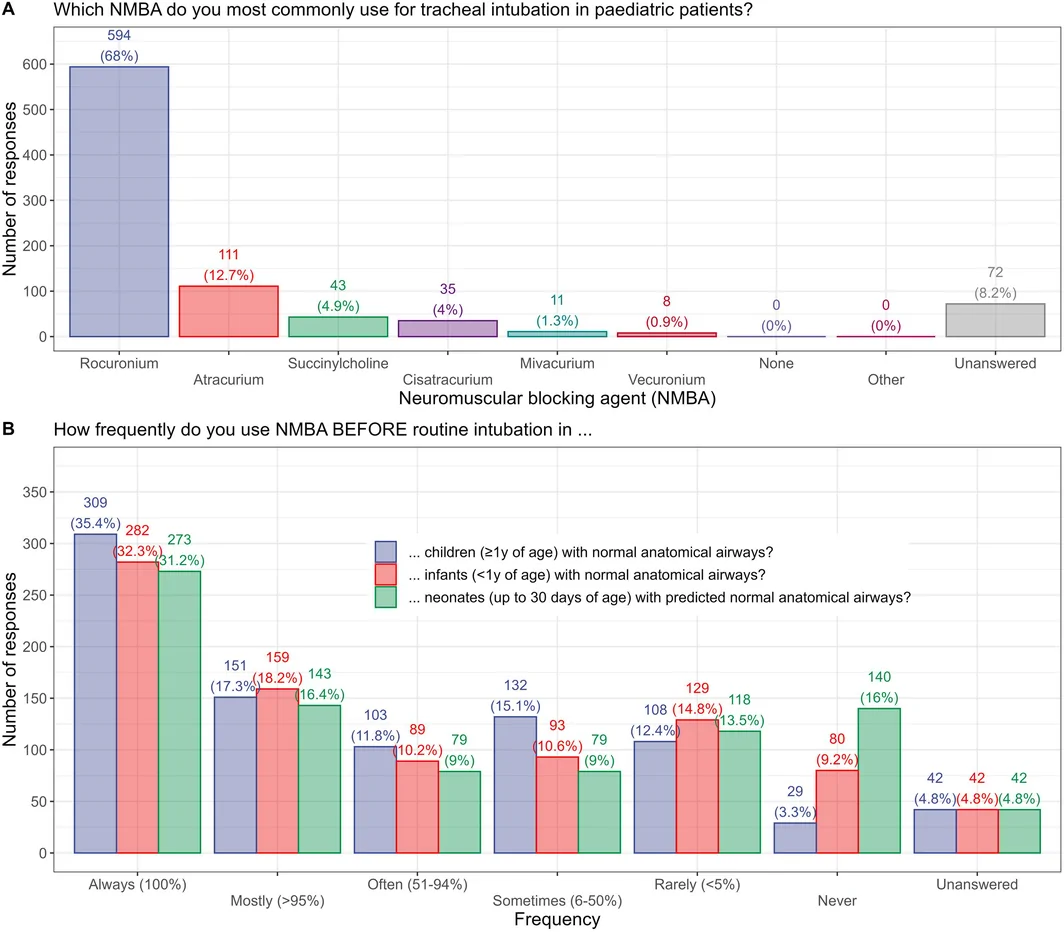
Self‐reported use of neuromuscular blocking agents (NMBA) and patterns of NMBA administration for different age groups with normal airways. Data are presented as number (percentage).

**TABLE 2:** Location of airway management, use of neuromuscular blocking agent (NMBA), and rapid sequence induction and intubation practice.

Characteristic	*N* = 874	95% CI
Where do you perform airway management? (multiple answers)		
Operating room	850 (97%)	96%, 98%
Non‐operating room	354 (41%)	37%, 44%
Emergency department	292 (33%)	30%, 37%
Intensive Care unit	242 (28%)	25%, 31%
Pre‐hospital	90 (10%)	8.4%, 13%
Which NMBA do you most commonly use for tracheal intubation in pediatric patients?		
Rocuronium	594 (68%)	65%, 71%
Mivacurium	11 (1.3%)	0.66%, 2.3%
Cis‐atracurium	35 (4.0%)	2.8%, 5.6%
Atracurium	111 (13%)	11%, 15%
Vecuronium	8 (0.9%)	0.43%, 1.9%
Succinylcholine	43 (4.9%)	3.6%, 6.6%
Other (specify)	0 (0%)	0.00%, 0.55%
Not answered	72 (8.2%)	6.5%, 10%
How frequently do you use NMBA before routine tracheal intubation in neonates (up to 30 days of age) with predicted normal anatomical airways?		
Always (100%)	273 (31%)	28%, 34%
Mostly (> 95%)	143 (16%)	14%, 19%
Often (51%–94%)	79 (9.0%)	7.3%, 11%
Sometimes (6%–50%)	79 (9.0%)	7.3%, 11%
Rarely (< 5%)	118 (14%)	11%, 16%
Never	140 (16%)	14%, 19%
Not answered	42 (4.8%)	3.5%, 6.5%
How frequently do you use NMBA before routine tracheal intubation in infants (< 1 year of age) with predicted normal anatomical airways?		
Always (100%)	282 (32%)	29%, 35%
Mostly (> 95%)	159 (18%)	16%, 21%
Often (51%–94%)	89 (10%)	8.3%, 12%
Sometimes (6%–50%)	93 (11%)	8.7%, 13%
Rarely (< 5%)	129 (15%)	13%, 17%
Never	80 (9.2%)	7.4%, 11%
Not answered	42 (4.8%)	3.5%, 6.5%
How frequently do you use NMBA before routine tracheal intubation in children (≥ 1 year of age) with predicted normal anatomical airways?		
Always (100%)	309 (35%)	32%, 39%
Mostly (> 95%)	151 (17%)	15%, 20%
Often (51%–94%)	103 (12%)	9.8%, 14%
Sometimes (6%–50%)	132 (15%)	13%, 18%
Rarely (< 5%)	108 (12%)	10%, 15%
Never	29 (3.3%)	2.3%, 4.8%
Not answered	42 (4.8%)	3.5%, 6.5%
How do you mainly confirm adequate neuromuscular block before tracheal intubation?		
Time interval after NMBA administration	487 (56%)	52%, 59%
Qualitative monitoring (visual or palpable)	42 (4.8%)	3.5%, 6.5%
Quantitative monitoring (TOF monitoring with ratio)	181 (21%)	18%, 24%
Clinical judgment	106 (12%)	10%, 15%
No confirmation	16 (1.8%)	1.1%, 3.0%
Not answered	42 (4.8%)	3.5%, 6.5%
Do you commonly perform bag‐mask ventilation during RSII?		
Yes, all children	302 (35%)	31%, 38%
Yes, children under 1 month	39 (4.5%)	3.2%, 6.1%
Yes, children under 1 year	64 (7.3%)	5.7%, 9.3%
Yes, children under 2 years	70 (8.0%)	6.3%, 10%
Yes, children under 12 years	63 (7.2%)	5.6%, 9.2%
Yes, children under 18 years	12 (1.4%)	0.75%, 2.5%
No	271 (31%)	28%, 34%
Not answered	53 (6.1%)	4.6%, 7.9%

*Note:* Data are presented as number (percentage) with 95% confidence intervals.

Abbreviations: NMBA, neuromuscular blocking agents; RSII, rapid sequence induction and intubation.

### 3.2 Neuromuscular Blocking Agents During Rapid Sequence Induction and Intubation

For RSII in children with a predicted normal anatomical airway, rocuronium was the most frequently selected agent (67%), while succinylcholine was selected by 24% of participants (Figure 3). Regarding ventilation strategy during RSII, 36.4% of respondents reported routinely performing bag‐mask ventilation in all children, whereas 31% reported not performing bag‐mask ventilation during RSII (Table 2). Age‐dependent approaches were frequently reported: 4.5% performed bag‐mask ventilation only in children younger than 1 month, 7.3% in those younger than 1 year, 8.0% in children younger than 2 years, and 7.2% in children under 12 years of age.

**FIGURE 3:**
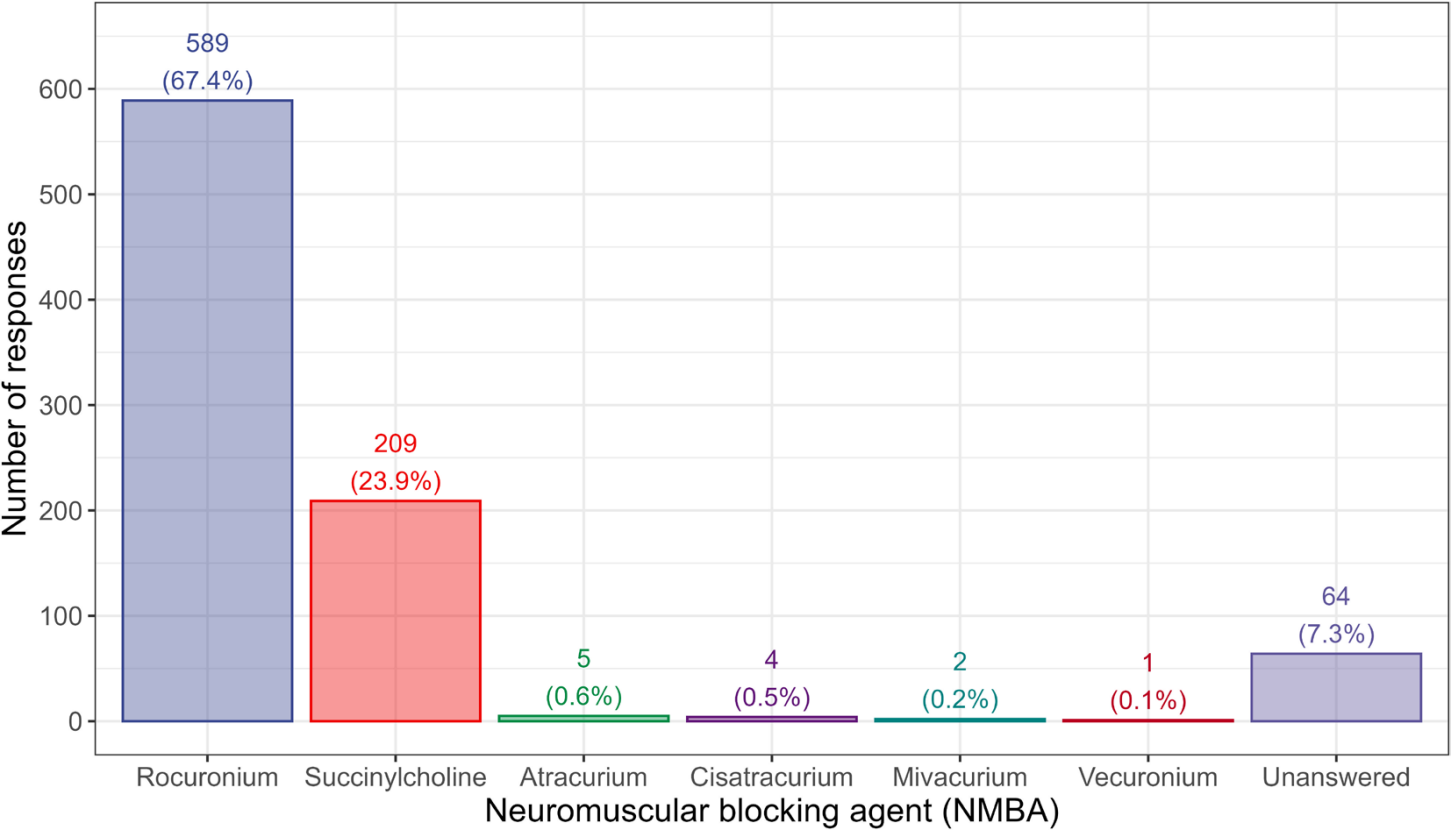
Self‐reported use of neuromuscular blocking agents (NMBA) for rapid sequence induction and intubation in pediatric patients with predicted normal anatomical airway at risk of pulmonary aspiration. Data are presented as number (percentage).

### 3.3 Confirmation of Adequate Neuromuscular Block Prior to Tracheal Intubation

When asked how an adequate level of neuromuscular block is assessed prior to tracheal intubation, most participants (56%) relied on a fixed time interval after NMBA administration. 21% of participants reported using quantitative monitoring (TOF ratio), while 4.8% opted for qualitative monitoring (visual or palpable assessment). Clinical judgment was reported as the sole method of assessment by 12% of participants, while a minority (1.8%) indicated that they did not assess the neuromuscular block prior to tracheal intubation (Table 2).

### 3.4 Availability and Use of Neuromuscular Monitoring

Availability of neuromuscular monitoring devices was high. A total of 61 participants (7.0%) reported access exclusively to qualitative monitors, 323 (37%) had access to quantitative monitors, and 326 (37%) had access to both device types. In contrast, 62 respondents (7.1%) reported no access to neuromuscular monitoring.

However, routine application was inconsistent. Overall, 152 participants (17%) reported using neuromuscular monitoring in over 95% of cases, while 127 (15%) reported infrequent use and 70 (8.0%) indicated that they never used monitoring (Table 3, Figure 2).

**TABLE 3:** Self‐reported neuromuscular block monitoring and reversal.

Characteristics	*N* = 874	95% CI
What type of neuromuscular monitoring devices are available in your institution?		
Qualitative	61 (7.0%)	5.4%, 8.9%
Quantitative	323 (37%)	34%, 40%
Both	326 (37%)	34%, 41%
None	62 (7.1%)	5.5%, 9.1%
Unsure	23 (2.6%)	1.7%, 4.0%
Not answered	79 (9.0%)	7.3%, 11%
What type of neuromuscular monitoring devices do you use in your clinical practice?		
Qualitative	78 (8.9%)	7.2%, 11%
Quantitative	382 (44%)	40%, 47%
Both	212 (24%)	21%, 27%
None	103 (12%)	9.8%, 14%
Unsure	20 (2.3%)	1.4%, 3.6%
Not answered	79 (9.0%)	7.3%, 11%
How often do you use neuromuscular block monitoring?		
Always (100%)	0 (0%)	0.00%, 0.55%
Mostly (> 95%)	152 (17%)	15%, 20%
Often (51%–94%)	122 (14%)	12%, 16%
Sometimes (6%–50%)	118 (14%)	11%, 16%
Rarely (< 5%)	127 (15%)	12%, 17%
Never	70 (8.0%)	6.3%, 10%
Not answered	285 (33%)	30%, 36%
At which age do you apply neuromuscular monitoring?		
> 1 month	91 (10%)	8.5%, 13%
> 1 year	110 (13%)	10%, 15%
> 3 years	149 (17%)	15%, 20%
Always	302 (35%)	31%, 38%
Never	143 (16%)	14%, 19%
Not answered	79 (9.0%)	7.3%, 11%
Preferred anatomic site for neuromuscular monitoring		
Arm (Adductor pollicis)	671 (77%)	74%, 80%
Head (Orbicularis oculi)	31 (3.5%)	2.5%, 5.1%
Leg (Posterior tibialis)	7 (0.8%)	0.35%, 1.7%
Other (specify)	0 (0%)	0.00%, 0.55%
Never use	79 (9.0%)	7.3%, 11%
Not answered	86 (9.8%)	8.0%, 12%
How often do you administer neuromuscular block reversal in children without contraindication?		
Always (100%)	240 (27%)	25%, 31%
Mostly (> 95%)	143 (16%)	14%, 19%
Often (51%–94%)	144 (16%)	14%, 19%
Sometimes (6%–50%)	154 (18%)	15%, 20%
Rarely (< 5%)	81 (9.3%)	7.5%, 11%
Never	25 (2.9%)	1.9%, 4.3%
Not answered	87 (10.0%)	8.1%, 12%
How do you commonly guide NMB reversal?		
Time interval after NMBA administration	120 (14%)	12%, 16%
Qualitative monitoring (visual or palpable)	77 (8.8%)	7.1%, 11%
Quantitative monitoring (TOF monitoring with ratio)	441 (50%)	47%, 54%
Clinical judgment	141 (16%)	14%, 19%
No guiding	8 (0.9%)	0.43%, 1.9%
Not answered	87 (10.0%)	8.1%, 12%
If quantitative monitoring (TOF monitoring with ratio)		
TOF ratio above 70%	41 (4.7%)	3.4%, 6.4%
TOF ratio above 80%	16 (1.8%)	1.1%, 3.0%
TOF ratio above 90%	108 (12%)	10%, 15%
TOF ratio at 100%	13 (1.5%)	0.83%, 2.6%
Not answered	696 (80%)	77%, 82%
Which reversal agents do you currently use in pediatric patients?		
Sugammadex	407 (47%)	43%, 50%
Neostigmine	103 (12%)	9.8%, 14%
Both	241 (28%)	25%, 31%
No reversal	16 (1.8%)	1.1%, 3.0%
Not answered	107 (12%)	10%, 15%
Does your institution restrict the use of sugammadex?		
Yes	87 (10.0%)	8.1%, 12%
No	680 (78%)	75%, 80%
Not answered	107 (12%)	10%, 15%
Do you personally limit sugammadex use because of cost or efficacy concerns?		
Yes—cost concerns	150 (17%)	15%, 20%
Yes—efficacy concerns	23 (2.6%)	1.7%, 4.0%
Yes—anaphylaxis	74 (8.5%)	6.7%, 11%
No concerns	518 (59%)	56%, 63%
Do you apply patient age restrictions for sugammadex use?		
No restriction	501 (57%)	54%, 61%
Restriction under 1 month	88 (10%)	8.2%, 12%
Restriction under 1 year	76 (8.7%)	7.0%, 11%
Restriction under 2 years	77 (8.8%)	7.1%, 11%
Restriction under 12 years	14 (1.6%)	0.91%, 2.7%
Restriction under 18 years	11 (1.3%)	0.66%, 2.3%
Not answered	107 (12%)	10%, 15%
Which dose of sugammadex do you administer most frequently for reversal of deep NMB (Train‐of‐four [TOF]): no detectable twitches (TOF count = 0 or post‐tetanic count [PTC]: 1–2 responses after a tetanic stimulus)?		
1 mg kg^−1^	21 (2.4%)	1.5%, 3.7%
2 mg kg^−1^	91 (10%)	8.5%, 13%
4 mg kg^−1^	444 (51%)	47%, 54%
8 mg kg^−1^	101 (12%)	9.6%, 14%
16 mg kg^−1^	110 (13%)	10%, 15%
Not answered	107 (12%)	10%, 15%
Which dose of sugammadex do you administer most frequently for reversal of moderate NMB (Train‐of‐four [TOF] count: 1–3 visible or palpable twitches)?		
1 mg kg^−1^	73 (8.4%)	6.6%, 10%
2 mg kg^−1^	480 (55%)	52%, 58%
4 mg kg^−1^	190 (22%)	19%, 25%
8 mg kg^−1^	19 (2.2%)	1.4%, 3.4%
16 mg kg^−1^	5 (0.6%)	0.21%, 1.4%
Not answered	107 (12%)	10%, 15%

*Note:* Data are presented as number (percentage) with 95% confidence intervals.

Abbreviations: NMB, neuromuscular block; PTC, post‐tetanic count; TOF, train‐of‐four.

When asked about age‐related use of neuromuscular monitoring, 35% of respondents (*n* = 302) reported applying monitoring across all pediatric age groups, including neonates. A further 40% (*n* = 350) reported using monitoring only from the age of 1 month onwards, while 16% (*n* = 143) indicated that they did not consider neuromuscular monitoring in pediatric patients.

### 3.5 Neuromuscular Block Reversal Practices

Reversal of neuromuscular block was administered “always” at the end of surgery by 27% of participants, and “mostly” by 16%. Conversely, 9.3% reported using reversal rarely and 2.9% never utilized reversal agents.

Among participants, 50% (441/874) guided reversal using quantitative neuromuscular monitoring, whereas 14% (*n* = 120) used a fixed time interval after NMBA administration, 16% (*n* = 141) relied on clinical judgment, and 8.8% (*n* = 77) used qualitative monitoring. Sugammadex was the predominant reversal agent (*n* = 407; 47%), while 28% of respondents (241) reported using both sugammadex and neostigmine as reversal agents. Only 16 respondents (1.8%) indicated that they did not use reversal agents at all (Table 3). For sugammadex, the doses most commonly administered were 4 mg kg^−1^ for deep neuromuscular block (PTC 1–2) and 2 mg kg^−1^ for moderate block (TOF count 1–3), as reported by more than 50% of participants.

## 4 Discussion

This international survey provides a contemporary overview of NMB management in pediatric anesthesia and intensive care. The findings reveal substantial practice variability across Europe and beyond, with four principal observations: (1) NMBA are not systematically administered prior to routine pediatric tracheal intubation, particularly in neonates; (2) NMBA choice differs markedly between routine tracheal intubation and RSII, with succinylcholine being still largely used (24%) during RSII; (3) quantitative NMB monitoring is widely available but markedly underutilized; and (4) NMB reversal is inconsistent and rarely guided by objective NMB monitoring.

### 4.1 Routine Tracheal Intubation Practices

The limited use of NMBA prior to routine pediatric tracheal intubation, particularly in neonates, probably reflects persisting clinician concerns about potential facemask ventilation difficulties and prolonged NMB in short procedures in this vulnerable population [11, 12]. Alternative strategies such as deep inhalational induction or short acting high‐dose opioid administration (e.g., remifentanil) may explain this pattern, yet the marked age‐dependency suggests insufficient standardization of pediatric airway management. Given evidence that NMBA use improves tracheal intubation conditions and reduces the risk of respiratory events and airway trauma at induction [1, 2, 3, 4, 5], this heterogeneity represents a priority area for patient safety improvement, unless spontaneous ventilation needs to be preserved for clinical reasons.

### 4.2 RSII Drug Selection and Ventilation Strategies

Rocuronium emerged as the preferred agent for routine tracheal intubation, consistent with its favorable profile in elective settings. However, succinylcholine remained entrenched in RSII practice (~25% preference), reflecting its rapid onset despite well‐documented risks including hyperkalemia in undiagnosed myopathies, bradycardia, and malignant hyperthermia triggering.

The survey also revealed substantial variability in RSII ventilation strategies: approximately one‐third of responders routinely ventilated all children during apnea, while another third avoided ventilation entirely. This dichotomy reflects the competing risks of hypoxemia versus aspiration, particularly given neonates' propensity for rapid desaturation. These findings underscore the need for pediatric‐specific RSII guidance that encompasses not only NMBA selection but also preoxygenation [13, 14] (a term coined to indicate oxygenation throughout the entire airway management period from preoxygenation, facemask ventilation, during laryngoscopy, until successful tracheal intubation), apneic oxygenation [15, 16], and controlled ventilation protocols.

### 4.3 NMB Monitoring Implementation Gap

The marked discrepancy between the availability of NMB monitors (> 80% of respondents) at the time of the survey and their routine clinical use (31%) underscores a critical patient safety concern. During induction, unmonitored conditions may lead to laryngoscopy before sufficient neuromuscular block, compromising tracheal intubation conditions and increasing aspiration risk during RSII. Even more concerning is the absence of objective monitoring at emergence, which precludes reliable detection of residual NMB, exposing children to postoperative airway obstruction, hypoventilation, and aspiration [8]. Only a few studies report on neuromuscular monitoring in pediatric patients [17], which reveals a gap of knowledge. Notably, the survey did not evaluate whether adequate NMB reversal was confirmed before extubation.

### 4.4 Inconsistent Reversal Practices

Over 10% of participants rarely/never administered reversal agents, and reversal decisions were infrequently guided by quantitative NMB monitoring. These findings highlight substantial variability in reversal practices and support the need for broader implementation of monitoring‐guided reversal strategies in pediatric patients. The reasons underlying this variability cannot be determined from the present survey but may include differences in institutional protocols, local practice culture, educational exposure, drug and equipment availability, and clinician beliefs regarding residual neuromuscular block. Reliance on clinical signs of recovery rather than objective monitoring also appears to remain common.

The inconsistent use of NMB monitoring and the inadequate reversal may expose children to avoidable postoperative respiratory complications, including airway obstruction, hypoventilation, impaired airway protective reflexes, aspiration, and the need for unplanned respiratory support [8]. In this context, the survey findings suggest that incomplete monitoring implementation represents a persistent patient safety gap, even in institutions where quantitative devices are available.

Finally, reversal practices are heterogeneous. More than one in ten participants reported rarely or never administering reversal agents in children, while reversal administration was not universally guided by quantitative monitoring. This combination of inconsistent monitoring and incomplete reversal represents a preventable safety issue with appropriate monitoring and reversal practises [8]. It may also reflect the absence of pediatric‐specific protocols, differences in institutional culture, and the persisting misconception that clinical recovery, for example, return of spontaneous ventilation, is sufficient to exclude residual paralysis.

A systematic review and meta‐analysis found that steroid NMBA induced NMB reversal with sugammadex is faster compared to neostigmine in children and adults, and might provide beneficial effects on postoperative nausea and vomiting [18]. A recent RCT investigating NMB reversal with sugammadex and neostigmine in 138 children under 2 years of age showed that 2 mg kg^−1^ for moderate and 4 mg kg^−1^ deep NMB with sugammadex were faster [19]. Survey participants reported the use of similar dosages.

### 4.5 Future Challenges and Research

Guideline implementation in pediatric anesthesia faces well‐recognized challenges. For instance, apneic oxygenation [15]—as recommended in BJA/ESAIC neonatal guidelines [3, 4]—remains underused, likely due to clinician unawareness [20]. This survey was intentionally conducted before publication of the 2025 ESAIC/ESPA pediatric NMBA guidelines [5] and therefore provides an important baseline against which future practice changes can be assessed. The marked variability observed in NMBA use, neuromuscular monitoring, and reversal practices highlights substantial opportunities for quality improvement and standardization of care.

Future work should focus not only on disseminating guideline recommendations but also on understanding and overcoming barriers to their implementation. Notably, our findings suggest that limited adoption of best practices cannot be explained solely by a lack of access to monitoring devices or reversal agents, as many respondents reported having these resources available yet using them inconsistently in clinical practice. Educational gaps, local practice culture, clinician beliefs regarding residual neuromuscular block, and the absence of institutional protocols may all contribute to the observed heterogeneity and warrant further investigation.

To evaluate the impact of the newly published guidelines, we plan to repeat this survey following guideline dissemination. Particular attention will be paid to changes in key metrics, including routine NMBA use for tracheal intubation, uptake of quantitative neuromuscular monitoring, monitoring‐guided reversal practices, and use of sugammadex. Future studies should also assess which implementation strategies, such as educational programs, local champions, protocol development, audit and feedback, or dedicated local pediatric airway leads [21], are most effective in promoting sustained adoption of guideline‐recommended practices.

Ultimately, improving pediatric neuromuscular block management will require more than the publication of evidence‐based recommendations. Success will depend on translating these recommendations into everyday clinical practice through targeted implementation efforts, continuous evaluation, and ongoing engagement of clinicians and institutions.

### 4.6 Limitations

This study has several limitations. Because the survey relied on self‐reported practice, it is subject to recall and reporting bias, and actual clinical behavior may differ from participants' responses. Voluntary participation may also have introduced selection bias, potentially over‐representing clinicians with a particular interest in pediatric airway management or neuromuscular block. The open‐dissemination strategy with mailing lists and social media prevented calculation of a precise response rate and limited the ability to assess non‐response bias. In addition, responses may reflect institutional resource availability rather than individual preferences, and variation within institutions could not be measured.

Additionally, the diverse clinical contexts and wide geographical scope contribute to response heterogeneity, which may limit generalizability to specific healthcare systems or practice settings.

Furthermore, the age categories used in some survey questions (> 1 month, > 1 year, and > 3 years) were defined pragmatically during questionnaire development rather than using a validated classification system. Although these thresholds reflect common developmental stages in pediatric anesthesia practice, alternative age groupings might have yielded different response patterns.

Despite these limitations, the multi‐society endorsement, large number of respondents, and broad geographical representation provide robust insights into current self‐reported pediatric NMBA practice patterns, highlighting actionable variability and key patient‐safety priorities.

## 5 Conclusion

Pediatric NMB management remains highly heterogeneous. A substantial proportion of clinicians continue to perform routine tracheal intubation without neuromuscular block, particularly in neonates. Marked differences also persist between routine tracheal intubation and RSII practices, including ongoing use of succinylcholine and wide variation in ventilation strategies. At the same time, quantitative neuromuscular monitoring and reversal strategies are applied inconsistently, despite availability of modern monitoring devices and effective reversal agents.

The most important finding from this survey is that the weakest link in pediatric NMB safety is not the availability of drugs or equipment. Many children are still intubated without NMBAs, monitored without objective tools, and extubated without reversal—or without confirmation of adequate reversal. These findings underscore an urgent need for implementation of the new pediatric neuromuscular block guidelines [4] at both national and local levels to ensure safe recovery from NMB across all pediatric age groups. The publication of dedicated pediatric guidelines represents a significant step toward more standardized clinical practice. However, meaningful improvement ultimately depends on successful translation of these recommendations into local clinical pathways, particularly given the wide spectrum of rare conditions within the pediatric population.

## Author Contributions

**Giulia Bonatti:** methodology, data curation, writing – original draft, writing – review and editing. **Mirjana Gander:** data curation, writing – original draft, writing – review and editing. **Arash Afshari:** data curation, writing – review and editing. **Francis Veyckemans:** data curation, writing – review and editing. **Thomas Fuchs‐Buder:** data curation, writing – review and editing. **Thomas Riva:** conceptualization, methodology, data curation, writing – original draft, writing – review and editing, supervision. **Markus Huber:** formal analysis, visualization. **Nicola Disma:** conceptualization, methodology, data curation, writing – original draft, writing – review and editing, supervision. **Alexander Fuchs:** conceptualization, methodology, data curation, writing – original draft, writing – review and editing, project administration, supervision.

## Funding

This investigator‐initiated web‐based cross‐sectional survey was solely financed with departmental grants from the Department of Anaesthesiology and Pain Medicine in Bern. The department had no influence on the conduct of the study or the interpretation of the data.

## Disclosure

We used AI‐assisted technologies (Grammarly PRO) to improve language, grammar, and readability. All corrections made by AI‐assisted technologies were approved by the authors.

## Conflicts of Interest

F.V.: Co‐Chairperson of the Syndromes and Rare Diseases in Pediatrics: Anesthesia website and a reviewer for OrphanAnaesthesia. T.F.‐B.: Receipt of honoraria or consultation fees: Merck. A.A.: Reviewer for OrphanAnaesthesia.

## Data Availability

The data that support the findings of this study are available from the corresponding author upon reasonable request.
